# Effects of exercise training on proteinuria in adult patients with chronic kidney disease: a systematic review and meta-analysis

**DOI:** 10.1186/s12882-020-01816-7

**Published:** 2020-05-11

**Authors:** Lei Yang, Xiaoxia Wu, Ying Wang, Chunfeng Wang, Rong Hu, Yong Wu

**Affiliations:** 1 School of Nursing, Fujian Medical University, No. 1 of Xueyuan Road, Shangjie Town, Minhou County, Fuzhou City, Fujian Province China; 2 Fujian Medical University Union Hospital, No. 29 of Xinquan Road, Fuzhou City, Fujian Province China

**Keywords:** Renal efficiency, Chronic, Proteinuria, Exercise, Meta-analysis

## Abstract

**Background:**

Rehabilitation effects of exercise training on adults with chronic kidney disease (CKD) have been generally recognised; however, the effects of exercise training on proteinuria have been underexplored. Our aim was to explore the effects of exercise training on proteinuria in adult CKD patients without renal replacement therapy.

**Methods:**

Randomised controlled trials (RCTs) and quasi-experimental studies examining the effects of exercise training on proteinuria in adults CKD patients without renal replacement therapy were searched in 10 electronic databases (MEDLINE, Embase, CINAHL, Cochrane Central Register of Controlled Trials, Allied and Complementary Medicine Database, SPORTDiscus with full text, Web of Science, China Wan Fang Database, China National Knowledge Internet, China Science and Technology Journal Database) until June 2019. The quality of quasi-experimental studies was assessed using the Joanna Briggs Institute Checklist for non-randomised experimental studies. The Cochrane risk of bias tool was used to evaluate the RCT quality.

**Results:**

We analysed 11 studies (623 participants). The 24-h urinary protein (24 h UP) level significantly decreased after exercise training in the within-group analysis (standard mean difference [SMD], 0.48; 95% confidence interval [CI], 0.08 to 0.88). There was a slight decrease in 24 h UP levels in the between-group analysis (SMD, 0.91; 95% CI, 0.00 to 1.82); however, the subgroup analysis showed that the change was insignificant (RCT: SMD, 0.24; 95% CI, − 0.44 to 0.92; quasi-experimental studies: SMD, 2.50; 95% CI, − 1.22 to 6.23). Exercise resulted in no significant differences in the urinary albumin-to-creatinine ratio in the between-group analysis (SMD, 0.06; 95% CI, − 0.54 to 0.67), but a significant decrease was found in the within-group analysis (SMD, 0.21; 95% CI, 0.04 to 0.38). No evidence of a decreased urinary protein-to-creatinine ratio was found after exercise (between-group analysis: SMD, 0.08 and 95% CI, − 0.33 to 0.48; within-group analysis: SMD, 0.04; 95% CI, − 0.25 to 0.32).

**Conclusion:**

Exercise training does not aggravate proteinuria in adult CKD patients without renal replacement therapy. Further research is warranted in the future to determine the effectiveness of exercise training on proteinuria and to explore the mechanisms by which exercise training influences proteinuria.

## Background

Proteinuria is a marker of renal damage and a predictor of the progress of chronic kidney disease (CKD) [1]. The 2012 guidelines for CKD explicitly mentioned the reduction of proteinuria as one of the markers of CKD staging [2]. The proteinuria level is an important predictor of disease progression, which is closely related to the occurrence of cardiovascular disease [3, 4]. Moreover, some studies [5–7] found that proteinuria can be used as a therapeutic target or endpoint [8] for the clinical treatment and prevention of cardiovascular complications, especially for patients with high proteinuria levels.

Exercise training has been recommended for patients with CKD by the Kidney Disease Improving Global Outcomes [2]. A substantial number of meta-analyses summarised the positive impacts of regular exercise programs for adults with CKD on physical performance, cardiopulmonary function, blood lipids, and quality of life [9–11]. A review suggested that high levels of physical activity appeared to be closely related to low proteinuria [12], and a cross-sectional study of non-diabetic women had similar results [13]. Afshinnia et al. [14] confirmed that exercise training can reduce proteinuria in obese people, although its long-term effect has not been confirmed by high-quality experimental studies. However, the sedentary time of patients with CKD, especially those with severe renal function impairment, is still significantly higher than that of individuals without CKD. Glavinovic et al. [15] reported that sedentary time of CKD was 10-times higher than that of individuals without CKD. Indeed, exercise is not a routine clinical treatment, and most CKD patients are worried about the safety of exercise, because sometimes high-intensity exercise can induce proteinuria [16]. A study has shown that strenuous exercise can increase the activity of the sympathetic nervous system and the blood concentration of catecholamine, thus increasing the permeability of glomerular capillary membrane, which leads to proteinuria [17]. Nevertheless, it seems that proteinuria returns to normal levels after 2 h of exercise [18].

No consensus has been achieved regarding the effect of exercise training on proteinuria in adult CKD patients without renal replacement therapy. Specific exercise programs for CKD are still being explored. Therefore, we conducted a systematic review and meta-analysis of randomised clinical trials (RCTs) and quasi-experimental studies to determine the effects of exercise training on proteinuria and to explore the effects of different exercise intensities on proteinuria in adult CKD patients without renal replacement therapy.

## Methods

### Protocol and registration

A systematic review was conducted according to a protocol registered at the International Prospective Register of Systematic Reviews (registration number CRD42019137192). This study followed the Preferred Reporting Items for Systematic Reviews and Meta-Analysis (PRISMA) guidelines [19, 20] and checklist (see Additional file 1).

### Search strategy

The Allied and Complementary Medicine Database, MEDLINE, Embase, and Cochrane Central Register of Controlled Trials were searched using Ovid SP. SPORTDiscus with full text and CINAHL were searched using the EBSCO host. A search of the Web of Science electronic databases (Science and Social Science Citation Index) was also conducted. Moreover, three Chinese databases, including the China Wan Fang Database, China National Knowledge Internet, and China Science and Technology Journal Database, were searched. The retrieval time was from the establishment of the database to June 2019.

By considering a broad range of phrases and terms used in the definitions related to CKD, exercise training, and proteinuria, we combined text words and Medical Subject Headings terms to search related terms, synonyms, and abbreviations. These include CKD, kidney insufficiency, chronic renal failure, exercise, physical activity, swimming, proteinuria, albuminuria, urinary albumin-to-creatinine ratio (UACR), and others. Furthermore, all references of the included studies were scanned manually to identify additional articles not found by our search. Only studies written in English or Chinese were included. The search strategy is outlined in Additional file (see Additional file 2).

### Study selection

**Fig. 1:**
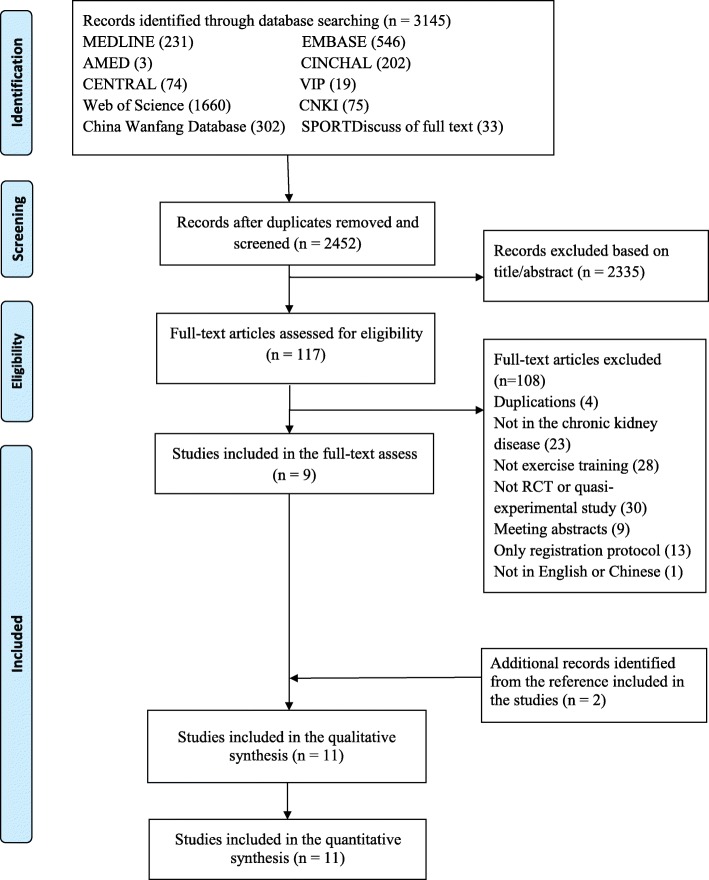
Flow diagram of the evaluation process

Two independent reviewers (L.Y. and X.W.) assessed the title or abstract according to the inclusion eligibility; if the abstract could not be determined, then the full text was screened. Disagreements during screening were resolved by consensus, and the final decision of the third reviewer (R.H.) was used if the consensus could not be achieved (Fig. 1). We included RCTs and quasi-experimental studies that reported one or more indicators of proteinuria both at baseline and after interventions.

The inclusion criteria were as follows: 1) participants were adult CKD patients without renal replacement therapy (18 years or older without kidney transplant and dialysis); 2) intervention included one or more modalities of regular exercise training, such as aerobic exercise, resistance exercise, and combined aerobic exercise and resistance exercise; 3) reported outcomes were one or more markers related to proteinuria, such as UACR, 24-h urinary protein (24 h UP), and urinary protein-to-creatinine ratio (UPCR); 4) control group with usual care or no exercise; and 5) the type of study included RCTs and quasi-experimental studies.

The exclusion criteria were as follows: 1) review or observational articles; 2) animal trials; and 3) non-English or non-Chinese articles.

### Data extraction

Data extraction was performed according to the prepared data selection forms created by two independent reviewers (L.Y. and X.W.). Any discrepancies were considered carefully and resolved through iteration and discussion. Data extracted included the following: 1) study characteristics, such as the year of publication, study design, sample size, and country; 2) description of the intervention, prescription of exercise program, modality, session length, intensity, frequency, setting, follow-up duration, supervised or not supervised, adherence reporting, measuring time point, and adverse event reported; 3) participant characteristics, such as body mass index, age, and related comorbidities/aetiology; and 4) reported outcomes including UACR, UPCR, and 24 h UP.

The primary outcome was the change in proteinuria, which was measured as a continuous variable. Given that the outcomes were measured at different times, we only extracted the endpoint of the intervention.

### Quality assessment

Each quasi-experimental study was evaluated for quality and risk of bias using the Joanna Briggs Institute Checklist for quasi-experimental studies [21], which includes nine items. The quality of RCTs was evaluated using the Cochrane Collaboration risk of bias checklist [22]. Any discrepancy concerning quality assessment was settled through a discussion.

### Data treatment and analysis

According to the American College of Sports Medicine [23], we classified exercise intensity as light, moderate, vigorous, and near maximal to maximal based on the physiological and perceived exertion responses.

Review Manager version 5.2 software (RevMan; the Cochrane Collaboration, Oxford, England; https://www.ncbi.nlm.nih.gov/pubmed/25450276) was used to analyse the data. The 95% confidence intervals (95% CIs) and standard mean differences (SMDs) for continuous data with inconsistent units, such as UACR (mg/mmol) and UACR (mg/g), were used. The data were pooled for meta-analysis when two or more trials measured the same outcome. For the trials reporting data as the median, interquartile range, or median and range, we translated these to the median and standard deviation for the meta-analysis. We only extracted the baseline data and data of the final follow-up period, although some trials reported data at more than one time. If a trial included a multiple intervention group without a shared control, then its data were entered separately. If there was a shared control, then we pooled the intervention groups using the proper formula from the *Cochrane Handbook for Systematic Reviews of Interventions* [24].

Heterogeneity was quantified using the I^2^ test, with I^2^ values of 25, 50, and 75% corresponding to low, moderate, and high levels of heterogeneity, respectively [25]. A subgroup analysis was used to determine whether the type of study leads to a potential heterogeneity (RCT, quasi-experimental study). A fixed effect model was used when heterogeneity was < 50%; otherwise, the random effect model was used. We tested if these studies would have changed the results through a sensitivity analysis. We did not test the publication bias of the included studies because the number for each outcome was too limited to perform funnel plots.

## Results

### Search results

Figure 1 presents a flow diagram of the included studies. We first searched 3145 studies from the 10 electronic databases. Of these, only 2452 studies remained after removing duplicates. Subsequently, we screened the title and abstract of these studies. The full texts of 117 potentially eligible studies were read. During the screening procedure, 108 articles were excluded at the full-text stage. Therefore, nine studies were eligible for inclusion after screening the full text. Furthermore, two studies were added after searching the reference list of the included studies. Finally, 11 studies were included in this review.

### Study characteristics

Table 1 provides a summary of the included studies. These were published between 2003 and 2019 in English and Chinese. Six were RCTs [26–31], of which two were pilot studies [29, 30]. Five were quasi-experimental studies [32–36], of which one was a single-arm trial [35]. Studies were conducted in the United States of America [29, 31, 34], Japan [30, 35], China [27, 36], Sweden [26], Estonia [32], Brazil [28], and England [33]; therefore, the data were from a variety of cultures.

**Table 1:** Characteristics of included studies

**Study**	**Study design**	**Simple size, n**	**Patients**	**Age, years**	**BMI, Kg/m**^**2**^	**Comorbidities/Etitology**	**Meds use: RAAS**	**Outcomes**	**Measuring time point**
Hellberg [26] Sweden (2019)	RCT	E1: 73	E1: CKD4–5	E1: 67 ± 14	E1: 28 ± 6	E1: DM/Hypertension	E: YES	U-ACR (mg/mmol)	0, 4, 8 mo
		E2: 75	E2: CKD4–5	E2: 65 ± 14	E2: 27 ± 5	E2: DM/Hypertension	C: YES		
Liang [27] China (2016)	RCT	E1: 29	E1: CKD2–3	E1: 48.21 ± 3.62	E1: 23.28 ± 2.49	E1: Hypertension	E1: YES	24 h UP (g/24 h)	0, 12 w
		E2: 29	E2: CKD2–3	E2: 48.50 ± 3.51	E2: 23.3 ± 2.53	E2: Hypertension	E2: YES		
		C: 29	C: CKD2–3	C: 48.00 ± 3.62	C: 23.25 ± 2.52	C: Hypertension	C: YES		
Aoike [28] Brizal (2017)	RCT	E1:12	E1: CKD3–4	E1: 56.0 ± 8.3	E1: 31.1 ± 4.6	E1: DM/Hypertension	E1: YES	Urinary protein (g/24 h)	0, 12, 24 w
		E2:13	E2: CKD3–4	E2: 56.3 ± 7.9	E2: 31.8 ± 4.5	E2: DM/Hypertension	E2: YES		
		C:15	C: CKD3–4	C: 54.3 ± 8.7	C: 30.7 ± 4.1	C: DM/Hypertension	C: YES		
Leehey [29] The USA (2009)	Pilot RCT	E:7	E: CKD2–4	E: NG	E: NG	E: DM	E: YES	UPCR (mg/g). UACR (mg/g)	0, 6, 24 w
		C: 4	C: CKD2–4	C: NG	C: NG	C: DM	C: YES	Urine protein excretion (mg/24 h)	
Hiraki [30] Japan (2017)	RCT	E: 14	E: CKD3–4	E: 69.0 ± 6.8	E: 24.4 ± 3.5	E: DM	E: NG	Urinary protein (g/gCr)	0, 12 mo
		C: 14	C: CKD3–4	C: 67.8 ± 6.9	C: 23 ± 2.5	C: DM	C: NG		
Leehey [31] The USA (2016)	RCT	E: 14	E: CKD2–4	E: 65.4 ± 8.7	E: 36.2 ± 4.8	E: DM2/Hypertension	E: YES	UPCR (mg/g)	0, 3, 13 mo
		C: 18	C: CKD2–4	C: 66.6 ± 7.5	C: 37.4 ± 4.2	C: DM2/Hypertension	C: YES	UACR (mg/g)	
Pechter [32] Estonia (2003)	Quasi-experimental study	E: 17	E: moderate CKD	E: 52 (31–72) *	E: 29.4 ± 1.3	E: Hypertension	E: NG	Urinary proteinuria excretion (U-Pro, g/24 h)	0, 12 mo
		C: 9	C: moderate CKD	C: 48 (35–65) *	C: 28.1 ± 1.3	C: Hypertension	C: NG		
Viana [33] England (2014)	Quasi-experimental study	E: 13	E: CKD4–5	E: 61 ± 8	E: 26.6 ± 4.7	E: NG	E: YES	UPCR (mg/mmol)	0, 6 mo
		C: 11	C: CKD4–5	C: 56 ± 16	C: 29 ± 5.9	C: NG	C: YES		
Nylen [34] The USA (2015)	Quasi-experimental study	E1: 38	E1: CKD1–3	E1: 62 ± 2.1	E1: NG	E1: DM2,	E1: NG	urinary albuminuria (UAE, mg/g creatinine).	0, 12 mo
		E2: 53	E2: CKD2	E:2: 62.5 ± 7.4	E2: NG	E2: DM2	E2: NG		
		E3: 37	E3: CKD3	E3: 63.8 ± 7.2	E3: NG	E3: DM2	E3: NG		
Hamada [35] Japan (2016)	A single-armed intervention study	E: 47	E: CKD1–5	E: 68.8 ± 11.8	E: 25.3 ± 3.8	E: DM	E: YES	Point of proteinuria: UPCR (g/gCr)	0, 6 mo
Zhang [36] China (2018)	Quasi-experimental study	E: 25	E: CKD2–3	E: 36.36 ± 10.12	E: NG	E: NG,	E: YES	Urinary proteinuria excretion (U-pro, mg/24 h)	0, 3, 6 mo
		C: 27	C: CKD2–3	C: 35.89 ± 9.64	C: NG	C: NG	C: YES		

Notes. E: Experimental group; C: Control group; RCT: Randomized controlled studies; CKD: Chronic kidney disease; BMI: Body mass index; NG: Not given; Med use: Medication use; RAAS: Renin-angiotensin-aldosterone system drugs; DM: Diabetes mellitus; DM2: Type II diabetes; UACR: Urinary albumin-to-creatinine ratio; 24 h UP: 24-h urinary protein; UPCR: Urinary protein-to-creatinine ratio; mo: month; w: week; *: median and range

### Patient characteristics

A total of 623 patients were allocated to the exercise training group (459) or no exercise group (164), with the sample size ranging from 13 to 148. Only two studies reported adherence [26, 30]. The mean age ranged from 35 to 69 years. The proportion of patients with a mean body mass index higher than 25 kg/m^2^ was 73%. Patients with hypertension [26, 28–30, 34, 35] or diabetes [26–29, 31, 32, 35] (together with CKD) were included in nine studies. Eight studies [26–29, 31, 33, 35, 36] reported the use of renin-angiotensin-aldosterone system drugs (RAAS), whereas the remaining three studies did not clearly report the drugs used [30, 32, 34].

### Exercise training characteristics

Studies in this review included all types of regular exercise training. Aerobic exercise was included as an intervention in all studies [26–36]. Resistance training, which was included in seven studies [26, 27, 30, 31, 34–36], was accompanied by aerobic exercise, leading to combined exercise training. In seven studies [26, 28, 30, 31, 35, 36], the exercise programs were conducted at home, at the park, or at the gym near the patients’ homes. In five studies [28, 29, 31, 33, 34], the exercise programs were conducted under supervision. Proteinuria was measured more than twice in five studies [26, 28, 29, 31, 36].

Training intensities were monitored using peak oxygen uptake in four studies [27–29, 31], the Borg rating of perceived exertion scale in five studies [26, 27, 30, 33, 35], metabolic equivalent in one study [35], heart rate reserve in one study [34], and international physical activity questionnaire in one study [36]. However, the tool used to monitor intensity in the remaining study was unclear [32, 36]. Of all the included studies, one study utilised low-intensity exercise training [32], six studies used moderate-intensity exercise training [27–30, 33, 35], and four studies used vigorous-intensity exercise training [26, 31, 34, 36]. The frequency of exercise training was three times or more per week in eight studies [26–31, 33, 36]. The highest exercise training frequency was five times per week [33]. Conversely, two studies used an exercise training frequency of less than three times per week [32, 35]. In one study, the frequency of exercise training was not reported adequately [34]. The duration of each session ranged from 30 to 120 min. The total follow-up duration ranged from 3 to 13 months. Eight studies had an exercise duration of more than 6 months [26, 29–31, 33–36]. However, the remaining three studies had an exercise duration of less than 6 months [27, 28, 32]. Exercise training details were outlined in Table 2.

**Table 2:** Exercise training parameters

**Study**	**Modality**	**Intensity**	**Session long, min**	**Follow-up, mo**	**Frequency**	**Setting**	**Supervision**	**Adherence reporting**
Hellberg [26] Sweden (2019)	E1: ST + ET	ST: RPE 13–17	ST: 90 min/w	12 mo	ST: 3 times/w	HB /nearby gym	NO	YES
	ET: Walking, running, cycling, and rowing etc.	ET: RPE 13–15	ET: 30 min, 60 min/w		ET: 2 times/w			
		(Vigorous)						
	ST: Quadriceps extension, hamstrings curl, lats-pull down, etc. 2–3 sets of 10 repetitions.							
	E2: BT + ET	ST: RPE 13–17	BT: 90 min/w	12 mo	BT: 3 times/w			
	ET: The same to E1.	BT: RPE 13–17	ET: 30 min, 60 min/w		ET: 2 times/w			
	BT: Static and dynamic balance exercises (maintaining balance while standing with feet together, standing on one leg, etc.) 10 repetitions, 2–3 sets.	(Vigorous)						
Liang [27] China (2016)	E1: AE	E1: 50%	AE: 30 min	3 mo	3 times/w	Unclear	Unclear	NO
	5 min warm up, ride a bike, 5 min of relaxation.	VO_2_peak RPE 12–13 (Moderate)	RE: 10 s/actions, 10 times/actions					
	E2: AE + RE	E2: 50% VO_2_peak						
	5 min warm up, ride a bike plus thera-band resistance training.	RPE 12–13						
		(Moderate)						
	C: High quality and low protein (≤0.6 g/kg/d) and other reasonable diet, and routine treatment.							
Aoike [28] Brizal (2017)	E1: HB AE AE: Walking.	E1: 40–60% VO_2_peak (Moderate)	AE: 30 min, Puls 10 min/4 w	3 mo	3 times/w	HB+ CB	YES	NO
	E2: CB AE AE: Walking on a treadmill.	E2: 40–60% VO_2_peak (Moderate)						
	C: Usual care							
Leehey [29] The USA (2009)	E: AE	E: 20–60% VO_2_peak	30 min	6 mo	3 times/w	CB+ HB	YES (6 w) NO (18 w) (Mixed)	NO
	AE: Walking on a treadmill. Warm-up, range-of-motion exercises, interval training, cool-down, and post-exercise range-of-motion exercises.	(Moderate)	Plus 5 min/2 w					
	C: Underwent the same testing battery but did not participate in any exercise training.							
Hiraki [30] Japan (2017)	E: AE + RE	E: RPE	AE: 30 min/8000	12 mo	3 times/w	HB	NO	YES
	AE: Walking.	(Moderate)	−10,000 steps					
	RE: Handgrip strengthening device squats and calf raises 20–30 repetitions per exercise.		RE: 20–30 min					
	C: Wore an accelerometer, but not given any exercise advice and continue the daily exercise.							
Leehey [31] The USA (2016)	E: AE + RE + diet (Nutritional counselling)	E: 25–84% VO_2_peak	AE: 60 min	13 mo	60 min/time- 3 times/w	HB	YES	NO
		(Vigorous)	RE: 20–30 min		30 min/time- 6 times/w			
	AE: Interval training on a treadmill.		HB (AE + RE):					
	RE: An elliptical trainer and cycle ergometer progressive resistance lower body exercise using elastic bands, hand-held weights or weight machine.		30 min/time, or 60 min/time					
	C: Diet (Nutritional counselling).							
Pechter [32] Estonia (2003)	E: AE	E: Low intensity	30 min	3 mo	2 times/w	Unclear	Unclear	N0
	AE: Water-based, vertically in the pool with total immersion (water temperature, 24 °C), 10 min warm-up exercises with gradually increasing intensity, 10 min cooling-down exercise.							
	C: Unclear							
Viana [33] England (2014)	E: AE	E: RPE 12–14	30 min	6 mo	5 times/w	HB	YES	NO
	AE: walking.	(Moderate)						
	C: Usual physical activity.							
Nylen [34] The USA (2015)	E1: AE + RE	E1: 50–80% HRR	60 min	3 mo	Unclear	Unclear	YES	NO
	AE + RE: 1 h session conclude that warm-up and cool-down and 30 min of combined aerobic and resistance training.	(Vigorous)						
	E2: The same to E1.	E2: 50–80% HRR	60 min	3 mo	Unclear			
		(Vigorous)						
	E3: The same to E1.	E3: 50–80% HRR	60 min	3 mo	Unclear			
		(Vigorous)						
Hamada [35] Japan (2016)	E: AE + RE	RE: 3–4 METS	90–120 min	6 mo	6 session/month	HB	Unclear	NO
	AE + RE: Resistance and effective walking.	AE: 12–14 RPE						
		(Moderate)						
Zhang [36] China (2018)	E:AE + RE	IPAQ	30 min	6 mo	3 times/w	HB	Unclear	NO
	Regular walking, yoga, aerobic gymnastics, biking, etc.	(Vigorous)						
	One or more forms of the aerobic and resistance exercise items.							
	C: Ordinary daily activities.							

Notes. *E* Exercise group, *C* Control group, *AE* Aerobic exercise, *RE* Resistance exercise, *w* week, *mo* month, *ST* Strength training, *BT* Balance training, *HB* Home-based, *CB* Center-based, *min* minutes, *HRR* Heart rate reserve, *ET* Endurance training, *RPE* Rating of perceived exertion, *METs* Metabolic equivalent, *VO*_*2*_*peak* Peak oxygen uptake, *IPAQ* International Physical Activity Questionnaire. The intensity of classification about low, moderate, and vigorous according to the advice of ACSM [23]

### Methodological quality

No study was excluded from the process of quality evaluation. The detailed quality assessment outlines are presented in Tables 3 and 4. However, the sample size in most studies was small, and the five articles were quasi-experimental studies; therefore, there may be a selection bias. Moreover, a majority of studies did not adequately report adherence.

**Table 3:** Quality evaluation of quasi-experimental studies

	Quasi-experimental studies				
Items	Pechter [32] (2003)	Viana [33] (2014)	Nylen [34] (2015)	Hamada [35] (2016)	Zhang [36] (2018)
1	Yes	Yes	Yes	Yes	Yes
2	Yes	Yes	Not applicable	Not applicable	Yes
3	Unclear	Yes	Yes	Yes	Unclear
4	Yes	Yes	Yes	Not applicable	Yes
5	No	No	No	No	Yes
6	Yes	Yes	Yes	Yes	Yes
7	Yes	Yes	Yes	Yes	Yes
8	Yes	Yes	Yes	Yes	Yes
9	Yes	Yes	Yes	Yes	Yes

Notes. 1 Is it clear in the study what is the ‘cause’ and what is the ‘effect’ (i.e. there is no confusion about which variable comes first)? 2 Were the participants included in any comparisons similar? 3 Were the participants included in any comparisons receiving similar treatment/care, other than the exposure or intervention of interest? 4 Was there a control group? 5 Were there multiple measurements of the outcome both pre and post the intervention/exposure? 6 Was follow up complete and if not, were differences between groups in terms of their follow up adequately described and analysed? 7 Were the outcomes of participants included in any comparisons measured in the same way? 8 Were outcomes measured in a reliable way? 9 Was appropriate statistical analysis used?

**Table 4:** Quality evaluation of randomised controlled trials

Randomised controlled studies						
Items	Hellberg [26] (2019)	Liang [27] (2016)	Aoike [28] (2017)	Leehey [29] (2016)	Hiraki [30] (2017)	Leehey [31] (2009)
1	Low	Low	Low	Low	Low	Low
2	Low	High	Unclear	Unclear	Unclear	Unclear
3	Low	High	Unclear	High	High	High
4	Low	Unclear	Unclear	Low	Low	Unclear
5	Low	Low	Low	Low	Low	High
6	Low	Low	Low	Low	Low	Low
7	Low	Low	Low	High	Low	High

Notes. 1 Random sequence generation (selection bias); 2 Allocation concealment (selection bias); 3 Blinding of participants and personnel (performance bias); 4 Blinding of outcome assessment (detection bias); 5 Incomplete outcome data (attrition bias); 6 Selective outcome reporting? (reporting bias) 7 Other bias

### Meta-analysis of exercise training and proteinuria

All studies reported indicators related to proteinuria. Four studies measured UACR [26, 29, 31, 34], five studies measured UPCR [29–31, 33, 35], and five studies reported 24 h UP [27–29, 32, 36]. Nine studies [26–30, 32–34, 36] presented the proteinuria data with mean values and standard deviations, and two studies [31, 35] used the median (range) score. Given the methodology heterogeneity of the included studies, a subgroup analysis of the study design was conducted.

### Between-group analysis

**Fig. 2:**
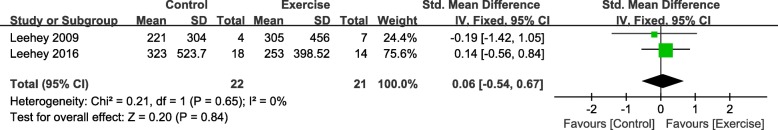
Change in UACR, Exercise vs. Control. Notes. 95% CI, 95% confidence interval; SMD, standardized mean difference; UACR, urinary albumin-to-creatinine ratio

We pooled two RCTs [29, 31] involving 43 participants that demonstrated a non-significant difference in the UACR between exercise training and control groups (SMD, 0.06; 95% CI, − 0.54 to 0.67; *P* = 0.84) (Fig. 2). No evidence of statistical heterogeneity was found (I^2^ = 0%).

**Fig. 3:**
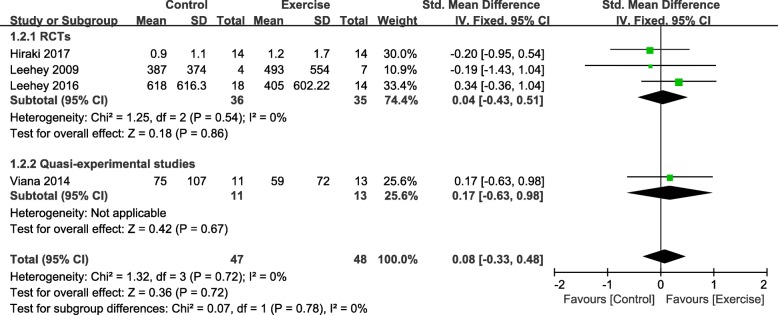
Change in UPCR, Exercise vs. Control. Notes. 95% CI, 95% confidence interval; SMD, standardized mean difference; UPCR, urinary protein-to-creatinine ratio

Four studies involving 95 participants reported UPCR [28–31, 33]. Synthesised data from four studies revealed a non-significant improvement in UPCR (SMD, 0.08; 95% CI, − 0.33 to 0.48; *P* = 0.72) (Fig. 3) for the exercise training and control groups, but no evident heterogeneity was seen for UPCR (I^2^ = 0%). There was no evidence of different effects on UPCR according to the different study designs (RCT: SMD, 0.04; 95% CI = -0.43 to 0.51, *P* = 0.86; quasi-experimental studies: SMD, 0.17; 95% CI = -0.63 to 0.98, *P* = 0.67).

**Fig. 4:**
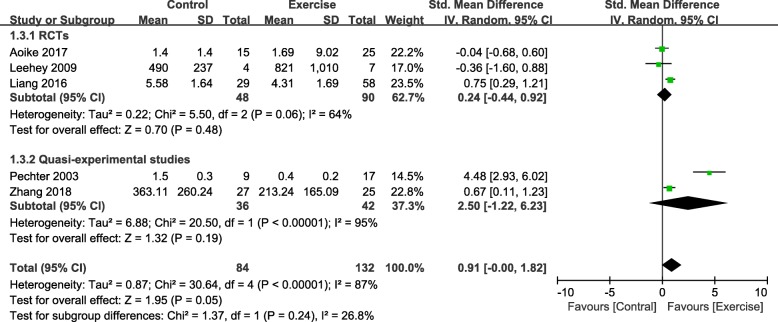
Change in 24 h UP, Exercise vs. Control. Notes. 95% CI, 95% confidence interval; SMD, standardized mean difference; 24 h UP, 24-h urinary protein

We pooled five studies [27–29, 32, 36] involving 216 participants that reported 24 h UP; the synthesised data suggested that there was a small significant decrease (SMD, 0.91; 95% CI, 0.00 to 1.82; *P* = 0.05) (Fig. 4) in 24 h UP. However, it should be noted that the statistical heterogeneity was substantial (I^2^ = 87%). There was a non-significant change in the 24 h UP of the RCTs and quasi-experimental studies (RCT: SMD, 0.24 and 95% CI, − 0.44 to 0.92, *P* = 0.48; quasi-experimental studies: SMD, 2.50 and 95% CI, − 1.22 to 6.23, *P* = 0.19).

### Within-group analysis

**Fig. 5:**
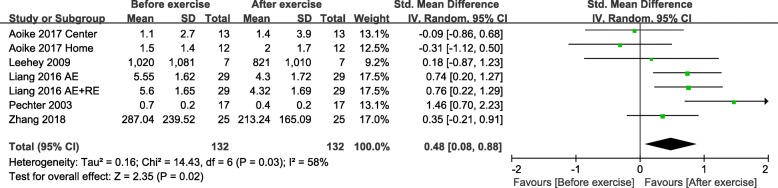
Change in 24 h UP, Before exercise vs. After exercise. Notes. 95% CI, 95% confidence interval; SMD, standardized mean difference; 24 h UP, 24-h urinary protein; AE, aerobic exercise; RE, resistance exercise

The change in 24 h UP from baseline was calculated from the five studies involving 132 participants in the exercise training group. Synthesised data revealed a significant decrease in 24 h UP (SMD, 0.48; 95% CI, 0.08 to 0.88; *P* = 0.02) (Fig. 5) in the exercise training group with moderate heterogeneity (I^2^ = 58%).

**Fig. 6:**
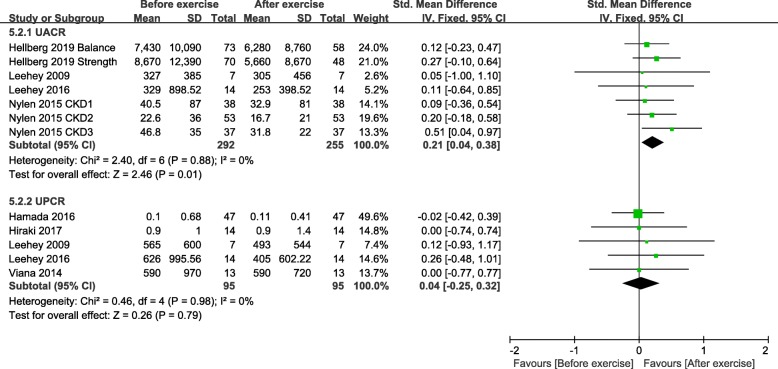
Change in UACR and UPCR, Before exercise vs. After exercise. Notes. 95% CI, 95% confidence interval; SMD, standardized mean difference; UPCR, urinary protein-to-creatinine ratio; UACR, urinary albumin-to-creatinine ratio

Four studies [26, 29, 31, 34] involving 292 participants in the exercise training group provided UACR data from baseline to the endpoint. In the RCT by Hellberg et al. [26] involving 148 participants, because there was no shared control group, we separated the data of the strength exercise and balance exercise groups, which were assessed as changes from baseline scores. The pooled data demonstrated a statistically significant decrease (SMD, 0.21; 95% CI, 0.04 to 0.38; *P* = 0.01) (Fig. 6). Statistical heterogeneity was not evident (I^2^ = 0%).

We synthesised five studies [29–33, 35] including 95 participants to explore the change in UPCR from baseline in the exercise training group. A non-significant change was observed following exercise training (SMD, 0.04; 95% CI, − 0.25 to 0.32; *P* = 0.79) (Fig. 6). The statistically significant heterogeneity detected was negligible (I^2^ = 0%).

### Narrative analysis of different exercise intensities and proteinuria

A low-intensity swimming exercise program [31] showed potential effects that could reduce proteinuria. In the six studies of moderate-intensity exercise, three studies [26, 28, 34] reported that there was a decreasing trend of proteinuria after exercise training; however, the remaining three studies [27, 29, 32] did not show a decreasing trend. In the four studies utilising vigorous-intensity exercise, one study by Viana et al. [33] reported that exercise did not change the proteinuria levels. However, the other three studies [25, 30, 35] showed a positive effect on the reduction of proteinuria, but it is worth noting that one study [30] was combined with dietary interventions. Moreover, attention should be focused on the fact that only a few of these studies yielded statistical significance, and the potential advantage was derived from the before exercise and after exercise comparison.

### Subgroup analysis results and sensitivity assessment

We conducted subgroup analyses according to the study design. Pooled SMD of RCTs indicated the non-significant effects of exercise training on UPCR [29–31] and 24 h UP [27–29]. Similarly, the pooled SMD of one quasi-experimental study [33] of UPCR and two of 24 h UP [32, 36] did not show significant effects. There was a difference in the study designs for exercise training and proteinuria, which may have been a potential cause of heterogeneity. In the sensitivity analysis, four studies [27, 32, 34, 36] could have been the source of heterogeneity, because removing these trials remarkably changed the results (see Additional file 3).

## Discussion

To the best of our knowledge, this is the first systematic review that assessed the relationship between exercise training and proteinuria. We found that exercise training did not aggravate proteinuria in adult CKD patients without renal replacement therapy, but the positive effects that could decrease proteinuria were uncertain. Exercises with intensity higher than moderate seemed to have the potential to reduce proteinuria, and low-intensity swimming training had a similar effect.

### Different exercise intensities and proteinuria

Evidence of the effects of low-, moderate-, and vigorous-intensity exercises was still inadequate during our assessment. Proteinuria levels decreased in CKD patients after 3 months of low-intensity swimming training [32]. However, we should note that the mechanism of swimming training is very different from that of other land exercises [37].

In all studies that implemented moderate and vigorous exercise programs, more than half of them (6/10) reported that proteinuria tended to decline. A previous review [38] reported that exercise could induce kidney damage, especially high-intensity exercise. Recently, some studies [39, 40] suggested that the risk of kidney damage increases only when the exercise intensity exceeded the lactic acid threshold. However, no adverse events related to exercise were reported in any of the included trials. Relative to studies are needed to resolve the discrepancies and further explore the effects of diverse exercise intensities on adult CKD patients without renal replacement therapy. Moreover, we found that the participants in six of the included studies [26, 27, 31, 34–36] well represented the CKD patients, whilst the participants in the remaining five studies [28–30, 32, 33] were strictly selected, such as including only male patients or those who had completed the stress, nutrition, and laboratory tests at the same time. Therefore, the conclusion may not be appropriate to the general CKD patients, the personal exercise programs with different intensities should be designed according to the physical function and disease status of the participants with CKD.

### Underlying mechanisms of exercise training and proteinuria

Although the mechanisms of exercise training and their effects on proteinuria are inconsistent, some hypotheses may explain the positive association. The production of proteinuria is associated with low inflammation and impaired endothelial function [41]. A potential mechanism is that the decrease in proteinuria level is potentially related to the reduction of hypersensitivity in C-reactive protein and IL-6 and the decrease of oxidative stress [42, 43]. An experimental study based on CKD that used combined spontaneous hypertension rates confirmed this view [44]. Moreover, exercise training has been shown to protect the vascular endothelial cells in cardiovascular patients [45], which is a crucial mechanism for low levels of proteinuria and a low incidence of cardiovascular disease in CKD patients. Furthermore, one author reported that aerobic exercise could significantly improve the levels of transforming growth factor beta and BB (platelet-derived growth factor BB) in CKD patients, thus contributing to the survival of CKD residual renal cells and fundamentally improving the kidney function of CKD patients, thereby reducing proteinuria [46].

Indeed, a decrease in blood pressure contributes to the reduction of proteinuria, potentially due to the decrease of renal hyperperfusion, high filtration rate, and selective permeability of the glomerular filtration membrane [2]. It is well known that RAAS drugs can effectively reduce the level of proteinuria whilst lowering the blood pressure [47]. In the included studies, more than 52% of the CKD patients were complicated by hypertension, but the exact number of patients taking RAAS drugs were not given clearly. It is worth noting that several studies [26–28, 35, 36] reported a decrease in blood pressure, but most studies did not analyze the effects of RAAS drugs and changes in blood pressure on proteinuria in detail, which might have caused confusion on whether there is a real exercise effect on proteinuria. Therefore, to confirm whether exercise has an effect on proteinuria, the factors involved in changes in proteinuria levels should be clarified.

These mechanisms seem to support that exercise training can reduce proteinuria, but the effect sizes reviewed in this study may not be clinically significant because the positive results are limited to their before exercise and after exercise comparison or are derived from the total effect of high heterogeneity. This may be because the sample size included in this study was too small, and the intervention time was not long enough to observe changes in proteinuria-related indicators.

### Expectations for the future

Our findings highlight several essential considerations for future studies. First, six studies monitored proteinuria only at baseline and at the end of the follow-up; however, because proteinuria is unstable [48, 49], it is necessary to continuously monitor proteinuria to ensure the authenticity of the data. Second, few studies reported compliance. It is common that the compliance of objects in exercise training to decline over time; therefore, participants may need to exercise under supervision rather than on their own. Future studies should specifically report exercise compliance along with the intensity and duration of exercise, completion of the treatment process, and changes in the health status of participants, including those who have not been followed up. A comprehensive report of this information will allow this essential variable to be included in future meta-analyses to confirm the effectiveness of exercise training interventions. Of note, the calculation of UACR is based on urine creatinine levels, which are influenced by increased muscle strength, protein intake, or decreased renal function [50]. We found that only a few studies [26, 32, 34, 36] have measured and analysed the change in muscle strength, volume, and creatinine levels whilst measuring UACR. To accurately evaluate whether exercise has a substantial effect on UACR, future studies should fully assess the impact of these potential factors.

### Advantages and limitations

This study had the key advantage of bibliographic database system retrieval, including the manual retrieval of citations, which provided a comprehensive search strategy and accounted for the potential defects of the database strategy. However, several limitations should be noticed when examining the results of our review. First, we included only published data and excluded the results of meeting abstracts and unpublished papers. Second, deviations from the historical controlled study may have led to the continued overestimation or underestimation of the effectiveness of the treatment. There may have been a selection bias due to the unpredictable differences between the two groups in the quasi-experimental study [51]. These deviations were large enough to cause research errors. Third, we could not conduct a subgroup analysis of exercise intensity because of the different types of studies included and the measurement of proteinuria; therefore, the conclusion regarding the effects of exercise at various intensities on proteinuria is uncertain. Finally, heterogeneity was only evaluated by the I^2^ test. However, the thresholds of I^2^ can be misleading because the importance of inconsistency is determined by several factors. We speculated that the source of heterogeneity would be the study design (RCTs and quasi-experimental studies), large differences in sample sizes (range, 13–148), and the exercise modality (swimming and land-based exercise). In addition, through a sensitivity analysis, we found that total effect value of 24 h UP was greatly affected by the two studies [27, 36]. We speculated that it may have come from the sample size of the two studies (87 and 52), which was larger compared with those of the other included studies; Nevertheless, the results in this study should be generalized with caution. Another source of heterogeneity may have been the proteinuria measurements because we know that the incidence of measurement error could be high for 24 h UP compared to that for spot proteinuria.

## Conclusion

Although the effects of the different exercise intensities on proteinuria are still unclear, exercise training with vigorous intensity is safe for adult CKD patients not receiving renal replacement therapy who have proteinuria. Further research is warranted in the future to determine the effectiveness of exercise training on proteinuria and to explore the mechanisms by which exercise training influences proteinuria.

## Supplementary information

**Additional file 1.** PRISMA 2009 Checklist.

**Additional file 2.** Appendix 1. Search strategy for each database.

**Additional file 3.** Appendix 2. Sensitivity analysis of 24 h UP, UACR and UPCR in between-group analysis and within-group analysis.

## Data Availability

All articles retained for this review were made available to the public through MEDLINE, Embase, CINAHL, Cochrane Central Register of Controlled Trials, Allied and Complementary Medicine Database, SPORTDiscus with full text, Web of Science, China Wan Fang Database, China National Knowledge Internet, China Science and Technology Journal Database. All data analysed in this study are included in the published articles.

## Abbreviations

CI: Confidence interval
CKD: Chronic kidney disease
RCT: Randomised controlled trial
SMD: Standard mean difference
UACR: Urinary albumin-to-creatinine ratio
UPCR: Urinary protein-to-creatinine ratio
24 h UP: 24-h urinary protein
E: Experimental group
C: Control group
BMI: Body mass index
NG: Not given
Med use: Medication use
RRAS: Renin-angiotensin-aldosterone system drugs
DM: Diabetes mellitus
DM2: Type II diabetes
mo: month
w: week
min: minutes
AE: Aerobic exercise
RE: Resistance exercise
ST: Strength training
BT: Balance training
HB: Home-based
CB: Center-based
HRR: Heart rate reserve
ET: Endurance training
RPE: Rating of perceived exertion
METs: Metabolic equivalent
VO_2_ peak: Peak oxygen uptake
IPAQ: International Physical Activity Questionnaire
