# Supplementary material for: Effects of exercise training on proteinuria in adult patients with chronic kidney disease: a systematic review and meta-analysis
Source: BMC Nephrol. 2020 May 11;21:172. doi: 10.1186/s12882-020-01816-7 (PMC7216591; doi:10.1186/s12882-020-01816-7)
Supplement: Supplementary file 2 — Additional file 2. Appendix 1. Search strategy for each database. [file 12882_2020_1816_MOESM2_ESM.docx]

**Appendix 1. Search strategy for each database**

| **Database** | **Search strategy** |
| --- | --- |
| via Ovid sp  Medline, 1946 to 4 June 2019 | 1. exp exercise movement techniques/ 2. exp exercise/ 3. ((resistance or aerobic$ or endurance$) adj3 exercise$). ab, ti. 4. ((exercise$ or resistance or strength or flexibility or endurance or pilates) adj6 (train$ or program$)). ab, ti.   5. (physical$ adj3 (activ$ or therap$ or exercise$)). ab, ti.  6. (dance therap$ or exercis$ or "tai ji" or "tai chi" or "tai-ji" or "tai-chi" or walking or yoga). ab, ti.  7. or /1—6.  8. exp Renal Insufficiency, Chronic/  9. (chronic kidney disease$ or kidney insufficiency or renal insufficiency or renal disease$). ab, ti.  10.(CKF or CKD or CRF or CRD). ab, ti.  11. (non-dialysis OR predialysis OR pre-dialysis), ab, ti. .  12. or / 11—14.  13. exp proteinuria/  14. (urinary albumin excretion or proteinuria$ or urinary protein or albuminuria or urinary albumin). af.  15. (UACR or UAER or ACR or PCR).af  16. (urine albumin-to-creatinine ratio or urine protein-to-creatinine ratio). af.  17. or/8-16.  18. 7 and 12 and 17. |
| via Ovid sp  Cochrane Central Register of Controlled Trials  (CENTRAL), 4 June 2019 | 1. exp exercise movement techniques/  2. exp exercise/  3. ((resistance or aerobic$ or endurance$) adj3 exercise$). ab, ti.  4. ((exercise$ or resistance or strength or flexibility or endurance or pilates) adj6 (train$ or program$)). ab, ti.  5. (physical$ adj3 (activ$ or therap$ or exercise$)). ab, ti.  6. (dance therap$ or exercis$ or "tai ji" or "tai chi" or "tai-ji" or "tai-chi" or walking or yoga). ab, ti.  7. or/1—6.  8. exp Renal Insufficiency, Chronic/  9. (chronic kidney disease$ or kidney insufficiency or renal insufficiency or renal disease$). ab, ti.  10. (CKF or CKD or CRF). ab, ti.  11. (non-dialysis or predialysis or pre-dialysis), ab, ti .  12.or/8-11.  13. exp proteinuria/  14. (urinary albumin excretion or proteinuria$ or urinary protein or albuminuria or  urinary albumin). af.  15. (UACR or UAER or ACR or PCR). af.  16. (urine albumin-to-creatinine ratio or urine protein-to-creatinine ratio). af.  17.or/13-16.  18. 7 and 12 and 17. |
| via Ovid sp  Embase (Classic + Embase), 1947 to 5 June 2019 | 1. exp exercise movement techniques/  2. exp exercise/  3. ((resistance or aerobic$ or endurance$) adj3 exercise$). ab, ti.  4. ((exercise$ or resistance or strength or flexibility or endurance or pilates) adj6 (train$ or program$)). ab, ti.  5. (physical$ adj3 (activ$ or therap$ or exercise$)). ab, ti.  6. (dance therap$ or exercis$ or "tai ji" or "tai chi" or "tai-ji" or "tai-chi" or walking or yoga). ab, ti.  7. or /1- 6.  8. exp Renal Insufficiency, Chronic/  9. (chronic kidney disease$ or kidney insufficiency or renal insufficiency or renal disease$). ab, ti.  10. (CKF or CKD or CRF or CRD). ab, ti.  11. (non-dialysis OR predialysis OR pre-dialysis), ab, ti .  12. or / 8-11.  13. exp proteinuria/  14. (urinary albumin excretion or proteinuria$ or urinary protein or albuminuria or urinary albumin or urine PRO). af.  15. (UACR or UAER or ACR or PCR). af  16. (urine albumin-to-creatinine ratio or urine protein-to-creatinine ratio). af.  17. or/13-17.  18. 7 and 12 and 17. |
| via Ovid sp  Allied and Complementary Medicine Database  (AMED), 1985 to 4 June 2019 | 1. exp exercise movement techniques/  2. exp exercise/  3. ((resistance or aerobic$ or endurance$) adj3 exercise$). ab, ti.  4. ((exercise$ or resistance or strength or flexibility or endurance or pilates) adj6 (train$  or program$)). ab, ti.  5. (physical$ adj3 (activ$ or therap$ or exercise$)). ab, ti.  6. (dance therap$ or exercis$ or "tai ji" or "tai chi" or "tai-ji" or "tai-chi" or walking or yoga). ab, ti.  7. or /1—6.  8. exp Kidney disease/  9. (chronic kidney disease$ or kidney insufficiency or renal insufficiency or renal disease$). ab, ti.  10. (CKF or CKD or CRF or CRD). ab, ti.  11. (non-dialysis or predialysis or pre-dialysis), ab, ti.  12. or /8-11.  13. (urinary albumin excretion or proteinuria$ or urinary protein or albuminuria or urinary albumin). af.  14. (UACR or UAER or ACR or PCR). af.  15. urine albumin-to-creatinine ratio or urine protein-to-creatinine ratio.  16. or/13-15.  17. 7 and 12 and 16. |
| via EBSCOhost  CINAHL complete, 5 June 2019 | S1 (MH "Exercise+") OR (MH "Therapeutic Exercise+") OR (MH "Recovery, Exercise") OR (MH "Exercise Test+") OR (MH "Exercise Intensity") OR (MH "Physical Activity") OR (MH "Sports+") OR (MH "Gait Training+")  S2 AB（exercise* OR resistance OR strength OR flexibility OR endurance）W6 ( train* OR program* )  S3 AB (physical* W3 (activ* OR therap* OR exercise*)  S4 AB (resistance OR aerobic* OR endurance*) W3 exercise*  S5 AB interval training OR sport* OR movement therapy*  S6 AB stretching  S7 AB dance therap* OR exercis* OR "tai ji" OR "tai chi" OR "tai-ji" OR "tai-chi" OR walking OR yoga  S8 OR /S1—S7  S9 (MH "Renal Insufficiency+")  S10 AB renal insufficienc* OR kidney insufficienc* OR renal diseas* OR kidney diseas*  S11 AB chronic kidney disease OR chronic renal insufficiency  S12 AB non-dialysis OR predialysis OR pre-dialysis  S13 AB CKF OR CKD OR CRF OR CRD  S14 OR/S19-S14  S15 (MH "Proteinuria+")  S16 TX proteinuria OR urinary protein OR albuminuria OR unrine albumin OR urinary albumin excretion  S17 TX UACR OR UAER OR ACR OR PCR  S18 TX urine albumin-to-creatinine ratio or urine protein-to-creatinine ratio  S19 OR/S15-18  S20 S8 AND S14 AND S19 |
| via EBSCOhost  SPORTDiscus of full text, 12 June 2019 | S1 (MH "Exercise+") OR (MH "Therapeutic Exercise+") OR (MH "Recovery, Exercise") OR (MH "Exercise Test+") OR (MH "Exercise Intensity") OR (MH "Physical Activity") OR (MH "Sports+") OR (MH "Gait Training+")  S2 AB（exercise* OR resistance OR strength OR flexibility OR endurance）W6 ( train* OR program* )  S3 AB (physical* W3 (activ* OR therap* OR exercise*)  S4 AB (resistance OR aerobic* OR endurance*) W3 exercise*  S5 AB interval training OR sport* OR movement therapy*  S6 AB stretching  S7 AB dance therap* OR exercis* OR "tai ji" OR "tai chi" OR "tai-ji" OR "tai-chi" OR walking OR yoga  S8 OR /S1—S7  S9 (MH "Renal Insufficiency+")  S10 AB renal insufficienc* OR kidney insufficienc* OR renal diseas* OR kidney diseas*  S11 AB chronic kidney disease OR chronic renal insufficiency  S12 AB non-dialysis OR predialysis OR pre-dialysis  S13 AB CKF OR CKD OR CRF OR CRD  S14 OR/S9-S13  S15 (MH "Proteinuria+")  S16 TX proteinuria OR urinary protein OR albuminuria OR urine albumin OR urinary albumin excretion  S17 TX urine albumin-to-creatinine ratio OR urine protein-to-creatinine ratio  S18 TX UACR OR UAER OR ACR OR PCR  S19 OR/S15-S18  S20 S8 AND S14 AND S19 |
| Web of Science (Science and Social Science Citation Index), 1945 to 5 June 2019 | #1 TS=" exercise movement techniques"  #2 TS="exercise therapy"  #3 TS= (exercise$ NEAR/3 resistance OR aerobic$ OR endurance$)  #4 TS= (exercise$ OR resistance OR strength OR flexibility OR endurance NEAR/6 train$)  #5 TS= (physical$ NEAR/3 activ$ OR therap$ OR exercise$)  #6 TS= (stretching OR dance therap$ OR exercise$ OR "tai ji" OR "tai chi" OR "tai-ji" OR "tai-chi" OR walking OR yoga)  #7 TS= (gymnastics OR running OR jogging OR swimming OR "cool-down exercise$" OR "endurance training" OR "warm-up exercise" OR "stair climbing")  #8 OR/1-7  #9 TS= ("kidney disease" OR "kidney insufficienc$" OR "renal insufficienc$" OR "renal disease" OR "chronic renal insufficienc$" OR "chronic kidney insufficienc$" OR "chronic kidney disease")  #10 TS= (CKF OR CKD OR CRF OR CRD)  #11 TS= (non-dialysis OR predialysis OR pre-dialysis)  #12 OR/9-11  #13 TS= (proteinuria OR albuminuria OR "urinary protein" OR "urinary albumin" OR "urinary albumin excretion ")  #14 TS= (UACR OR UAER OR ACR OR PCR)  #15 TS= (urine albumin-to-creatinine ratio OR urine protein-to-creatinine ratio)  #16 OR/13-15  #17 TS= ("randomized controlled trial" OR "controlled clinical trial" OR randomized OR placebo OR randomly OR trial OR quasi-experiment$ OR CCT OR RCT)  #18 #20 AND #8 AND #12 AND #16  #19 #18 AND Language: (English)  #20 #19 AND Language: (Chinese)  #21 #19 OR #20 |
| China National Knowledge Infrastructure (CNKI),  1979 to 5 June 2019 | SU= ('exercise' OR 'exercise training' OR 'exercise prescription' OR 'exercise therapy' OR 'exercise intervention' OR 'physical activity' OR 'aerobic exercise' OR 'anaerobic exercise' OR 'resistance exercise' OR 'resistance training' OR 'strength' OR 'aerobic endurance' OR 'exercise modality') AND SU= ('chronic kidney disease' OR 'kidney disease' OR 'chronic renal deficiency' OR 'chronic renal function deficiency' OR 'chronic renal kidney deficiency' OR 'chronic renal disease') AND FT= ('proteinuria' OR ' urine protein' OR 'urinary protein-to-creatinine ratio' OR ' urinary-albumin- excretion ratio ' OR '24-hour urinary protein' OR 'UACR' OR 'ACR' OR 'UPCR' OR '24h UP') |
| China Wan Fang Database,  1977 to 5 June 2019 | SU (exercise OR exercise therapy OR exercise prescription OR physical activity OR exercise training OR exercise intervention OR aerobic exercise OR anaerobic exercise OR resistance training OR strength OR aerobic endurance OR exercise modality) AND SU (chronic kidney disease OR kidney disease OR chronic renal deficiency OR chronic renal function deficiency OR chronic renal disease OR chronic renal deficiencies) AND SU (proteinuria OR urine protein OR 24-hour urine protein OR urinary protein-to-creatinine ratio OR urinary albumin-to-creatinine ratio OR urinary albumin excretion rate OR UACR OR ACR OR 24h UP OR UPCR) |
| Journal Integration Platform (VIP),  1989 to 5 June 2019 | R= ( exercise OR exercise intervention OR exercise prescription OR exercise therapy OR exercise training OR physical activity OR aerobic exercise OR anaerobic exercise OR endurance exercise OR strength OR aerobic endurance OR resistance exercise OR resistance training OR exercise therapy OR ) AND R= (chronic kidney disease OR renal disease OR chronic renal insufficiency OR chronic kidney deficiency OR chronic renal disease OR chronic renal function deficiency）AND U= (proteinuria OR urine protein OR 24-hour urine protein OR urinary albumin-to-creatinine ratio OR urinary protein-to-creatinine ratio OR urinary albumin excretion rate OR UPCR OR UACR OR ACR OR 24h UP) |

Notes: ab=abstract; ti=title; af=any field; AB=abstract; MH=MeSH; TS=subject; TX=full text; SU=subject; R=abstract; FT=full text; U=any field

RCT=randomised controlled trial; CCT=controlled clinical trail
