# Supplementary material for: Effects of exercise training on proteinuria in adult patients with chronic kidney disease: a systematic review and meta-analysis
Source: BMC Nephrol. 2020 May 11;21:172. doi: 10.1186/s12882-020-01816-7 (PMC7216591; doi:10.1186/s12882-020-01816-7)
Supplement: Supplementary file 3 — Additional file 3. Appendix 2. Sensitivity analysis of 24 h UP, UACR and UPCR in between-group analysis and within-group analysis. [file 12882_2020_1816_MOESM3_ESM.docx]

**Appendix 2. Sensitivity analysis of 24h UP, UACR and UPCR in between-group analysis.**

| Study removed | SMD (95% CI) | *P*-value | Study removed | SMD (95% CI) | *P-*value | Study removed | SMD (95% CI) | *P-*value |
| --- | --- | --- | --- | --- | --- | --- | --- | --- |
| 24h UP |  | | UACR |  |  | UPCR |  |  |
| Aoike (2017) | 1.21 [0.07, 2.35] | 0.04 | Leehey (2009) | 0.14 [-0.56, 0.84] | 0.40 | Hiraki (2017) | 0.20 [-0.29, 0.68] | 0.43 |
| Leeheyl (2009) | 1.18 [0.16, 2.20] | 0.02 | Leehey (2016) | -0.19 [-1.42, 1.05] | 0.77 | Leehey (2009) | 0.11 [-0.32, 0.54] | 0.62 |
| Liang (2016) | 1.04 [-0.33, 2.42] | 0.14 |  |  |  | Leehey (2016) | -0.06 [-0.56, 0.44] | 0.82 |
| Pechter (2003) | 0.40 [-0.06, 0.86] | 0.09 |  |  |  | Viana (2014) | 0.04 [-0.43, 0.51] | 0.86 |
| Zhangl (2018) | 1.05 [-0.26, 2.36] | 0.12 |  |  |  |  |  |  |

Notes. UACR: Urinary albumin-to-creatinine ratio; 24h UP: 24-hour urinary protein; UPCR: Urinary protein-to-creatinine ratio; 95% CI: 95% confidence interval; SMD: Standardized mean difference.

**Appendix 2. Sensitivity analysis of 24h UP, UACR and UPCR in within-group analysis.**

| **Study removed** | **SMD (95% CI)** | ***P*-value** | **Study removed** | **SMD (95% CI)** | ***P*-value** | **Study removed** | **SMD (95% CI)** | ***P*-value** |
| --- | --- | --- | --- | --- | --- | --- | --- | --- |
| **24h UP** |  | | **UACR** |  |  | **UPCR** |  |  |
| Aoike (2017) Center | 0.57 [0.15, 0.99] | 0.008 | Hellberg (2019) Strength | 0.20 [0.01, 0.39] | 0.04 | Hamada (2016) | 0.09 [-0.31, 0.49] | 0.65 |
| Aoike (2017) Home | 0.60 [0.22, 0.98] | 0.002 | Hellberg (2019) Balance | 0.24 [0.05, 0.44] | 0.01 | Hiraki (2017) | 0.04 [-0.26, 0.35] | 0.78 |
| Leehey (2009) | 0.51 [0.07, 0.95] | 0.02 | Leehey (2009) | 0.22 [0.04, 0.39] | 0.01 | Leehey (2009) | 0.03 [-0.26, 0.33] | 0.83 |
| Liang (2016) AE | 0.42 [-0.06, 0.90] | 0.09 | Leehey (2016) | 0.22 [0.04, 0.39] | 0.01 | Leehey (2016) | -0.00 [-0.31, 0.31] | 1.00 |
| Liang (2016) AE+RE | 0.42 [-0.06, 0.90] | 0.09 | Nylen (2015) CKD1 | 0.23 [0.05, 0.41] | 0.01 | Viana (2014) | 0.04 [-0.26, 0.35] | 0.78 |
| Pechter (2003) | 0.36 [0.02, 0.71] | 0.04 | Nylen (2015) CKD2 | 0.22 [0.03, 0.40] | 0.03 |  |  |  |
| Zhang (2018) | 0.50 [0.01, 0.98] | 0.04 | Nylen (2015) CKD3 | 0.17 [-0.02, 0.35] | 0.07 |  |  |  |

Notes. UACR: Urinary albumin-to-creatinine ratio; 24h; UP: 24-hour urinary protein; UPCR: Urinary protein-to-creatinine ratio; CKD: Chronic kidney disease; 95% CI: 95% confidence interval; SMD: Standardized mean difference; AE: Aerobic exercise; RE: Resistance exercise.
